# Both plant genotype and herbivory shape aspen endophyte communities

**DOI:** 10.1007/s00442-018-4097-3

**Published:** 2018-03-01

**Authors:** Benedicte Riber Albrectsen, Abu Bakar Siddique, Vicki Huizu Guo Decker, Martin Unterseher, Kathryn M. Robinson

**Affiliations:** 1 Department of Plant Physiology, Umeå University, Umeå Plant Science Centre, Umeå, Sweden; 2 Ernst-Moritz-Arndt Universität Greifswald, Institut für Botanik und Landschaftsökologie, Soldmannstr. 15, 17487 Greifswald, Germany; 3 Evangelisches Schulzentrum Martinschule, Max-Planck- Str. 7, 17491 Greifswald, Germany

**Keywords:** Herbivory, Arboreal endophytes, Salicinoid, Bipartite graphics, Competition

## Abstract

Salicinoid phenolic glycosides are common defence substances in salicaceous trees and specialist leaf beetles use these compounds for their own defence against predators. Salicinoids vary qualitatively and qualitatively in aspen (*Populus tremula*) and this variation has a genetic basis. The foliar endophyte mycobiome is plentiful and we hypothesised that it is related to plant genotype, potentially mediated by salicinoid composition, and that interactions with the leaf beetle *Chrysomela tremula* may alter this relationship. We studied these three-way interactions in controlled greenhouse experiments. Endophytic fungi were isolated from sterilised leaf tissues with and without beetle damage, and from beetles. We confirmed that endophyte composition was influenced by host genotype. Beetle activity added generalist morphs to the mycobiome that overrode the initial host association. Yeast-like genera (*Cryptococcus* and *Rhodotorula*) were isolated only from beetle-damaged tissues and from beetles, whereas fast-growing filamentous fungi dominated beetle-free control plants. Competition experiments between filamentous fungi of plant origin and beetle-related yeasts suggested interaction of both stimulating and inhibiting modes of action amongst the fungi. As a result, we detected examples of amensalism, commensalism, parasitism and competition between the morphs tested, but we found no evidence of mutualism, and consequently no co-evolutionary relationship could be demonstrated, between yeasts carried by beetles, host genotype and associated filamentous morphs. Endophyte studies are method-dependent and high-throughput sequencing technology best define the fungal mycobiome, culturing however continues to be a cheap way to provide fundamental ecological insights and it is also required for experimental studies.

**Electronic supplementary material:**

The online version of this article (10.1007/s00442-018-4097-3) contains supplementary material, which is available to authorized users.

## Introduction

Horizontally transferred fungal endophytes live hidden lives in plants (Petrini and Fisher 1990; Hyde and Soytong 2008). Composition and abundance vary among tissues (Partida-Martínez and Heil 2011), in their host specificity, and by environment (e.g. Carroll 1995; Elamo et al. 1999; Santamaria and Diez 2005; Martín-García et al. 2011; Albrectsen et al. 2010a; Unterseher et al. 2016; Lamit et al. 2014; Siddique and Unterseher 2016). Increasing evidence also suggests that endophytes can manipulate consumers of their host (e.g. Herre et al. 2007; Gange et al. 2012; Hammer and Van Bael 2015; Hiscox and Boddy 2017) supporting the hypothesis that host–endophyte interactions may be mutualistic (Schulz and Boyle 2005; Saikkonen et al. 1998).

Defensive mutualism has been attributed to systemic endophytes (*Epichloë* spp.) in cold season grasses (Clay 1988), whereas endophytes of woody plants are considered non-systemic random dispersers that establish when they encounter appropriate hosts (Saikkonen et al. 1996, 1998, 2007; Sieber 2007; Rodriguez et al. 2009). Since trees typically have long generation times and slow rates of molecular evolution, it has been proposed that the presence of a diverse mycobiota could add a layer of phenotypic diversity, which might “assist” the tree in manipulating its enemies (Albrectsen and Witzell 2012; Christian et al. 2017). In support of this, rust infections became less severe in *Populus* leaves that had previously been inoculated with certain endophytes (i.e. belonging to *Cladosporium*, *Penicillium*, *Tricoderma* and *Chaetomium*; Raghavendra and Newcombe 2013; Busby et al. 2016), and gall-forming cynipid wasps on oak avoided to oviposit on leaf parts that were prone to endophyte colonisation (Wilson and Carroll 1997). Moreover, in support of endophyte anti-herbivore effects, galling aphids appear to alter and proliferate the endophytic mycobiota surrounding their galls (Lawson et al. 2014), and leaf cutting ants clean their leaves of endophytes before they plant them in their fungal garden (Van Bael et al. 2009; Coblentz and van Bael 2013).

However, insect herbivores and endophytes can also be positively correlated (Wilson and Faeth 2001), or they may negatively affect higher order parasitism (Preszler et al. 1996), both resulting in an overall negative (neutral to antagonistic) effect on the host plant. Endophytes may thus be essential for ecosystem functioning (Arnold 2007; Wilson 2000; Albrectsen and Witzell 2012; Unterseher et al. 2016). Moreover, like pathogens, the endophytes must overcome protective barriers produced by the host (Petrini and Fisher 1990; Lappalainen and Helander 1997; Elamo et al. 1999; Santamaria and Diez 2005; Bailey et al. 2005), which indicates that at least some endophytes may have evolved from plant pathogens (Vega and Dowd 2005). Association with animal life further suggests that other endophytes evolved from entomopathogens (Graham 1967; Vega and Dowd 2005).

Twig endophytes in hybrid cottonwoods correlate with condensed tannin concentrations (Bailey et al. 2005) but not with the content of salicinoid compounds. Black yeasts *(Aureobasidium* spp.) in *Populus tremula* have also been related to the presence of phenolic compounds across growing environments (Albrectsen et al. 2010a), and salicinoids deter generalist herbivores from eating leaves of *P. tremuloides* (Donaldson and Lindroth 2007). Salicinoids on the other hand attract specialist herbivores such as leaf beetles (Chrysomelidae*)* that can use the compounds for their own protection (e.g. Pasteels et al. 1983; Termonia et al. 2001; Boland 2015). In fact, most of the arthropods that feed on aspen are specialists (Robinson et al. 2012) but the relationships with the foliar phenolic profile in field studies are mostly weak (Albrectsen et al. 2010b). Salicinoid phenolic profiles are relatively heritable and stable within genets and across environments (Keefover-Ring et al. 2014). Environmental factors such as nitrogen addition, however, affect the biosynthesis of most phenolic classes (Decker et al. 2017). None of these factors systematically associates with any attacker, but host growth influences the composition of arthropod communities (Robinson et al. 2012).

*Chrysomela tremula* leaf beetles are plentiful on juvenile aspen suckers (Robinson et al. 2009). Due to their preference for salicinoid compounds, we hypothesised that these beetles would directly or indirectly interfere with the endophyte community in the foliar endosphere of *P. tremula*, which we initially expected to be associated with genotype (Albrectsen et al. 2010a, b). Furthermore, we looked for evidence of competitive relationships between fungi of, respectively, plant and potential herbivore origin.

## Materials and methods

### Plant material

For this study, we used eight aspen genotypes (genets) from the SwAsp collection (Luquez et al. 2008; 7, 14, 18, 23, 52, 60, 65, 100). The SwAsp individuals have been categorized based on foliar salicinoid composition (Keefover-Ring et al. 2014) that varies qualitatively and quantitatively with genotype, and of which ca. 40% of the SwAsp collection produces predominantly salicin, salicortin, tremulacin, and tremuloidin, analogous to the salicinoid profiles reported in *P. tremuloides* (Lindroth et al. 1988, see also Keefover-Ring et al. 2014). Additional salicinoids in *P. tremula* have characteristic functional groups adhered to the glucose molecule, for example a cinnamoyl moiety, which is present at detectable levels in ca. 50% of the SwAsp collection but undetected in *P. tremuloides*. The genets used in this experiment can be classified by the production of the cinnamoyl moiety: genotypes 7, 18, 60, 100 are tremuloides-like (TL), and genotypes 14, 23, 52, 65 produce salicinoids with cinnamoyl moieties (CN).

### Beetle material

A colony of *Chrysomela tremula* was established in the summer 2011, using leaf beetles originating from one site of recently cut aspens at Mariehem, Umeå (63.844879°N, 20.339819°E). Five hundred beetles representing both larval and adult stages (a minimum of 200 adults) were reared in the laboratory on a diet of mixed *P. tremula* genotypes. The mixed diet and mixed larval/adult stages were used to try to avoid effects of diet that might otherwise have caused conditioning of certain aspen genets for feeding or oviposition preference. An even sex ratio was considered (Hamilton 1969), and no attempt was made to separate the herbivores according to sex. To obtain information about the fungi that the beetles might introduce to the plants, surface-sterilised beetles (using the same procedure as for leaves) were left on PDA plates to detect the fungi they might host.

### Isolation of cultivable endophytes

Cultivable fungal endophytes were isolated from leaves as follows. Fully expanded leaves similar to those harvested for chemistry were picked and first submerged in 70% ethanol for 5 s, then in 2% sodium hypochlorite for 4 min and thereafter rinsed twice in distilled water for 10 s. Under the laminar hood (KOJAIR, 0.50 m/s airflow) and under sterile conditions, leaves were then cut into ca. 1 cm^2^ pieces that were placed on PDA plates. Samples were incubated in a growth chamber (Leec) and the colonies that appeared on the plates were described and their growth assessed every second day for 2 weeks. Fungal colonies were morphotyped on the basis of colour, morphology, and growth patterns (ESM1).

### The experiments

Two fungal isolation experiments were conducted. The first one was performed with a full setup of eight genotypes. The second experiment with only two genotypes (18, 65) was performed to confirm initial patterns of association with genotype and beetle effects on richness and abundance of fungal morphs.

*Experiment 1.* The aspen genets were propagated from in vitro culture, and on October 7th 2011 transferred to soil with K-jord potting compost (Hasselfors Garden, Örebro, Sweden) in 1-L pots. The plants were grown in the greenhouse (Umeå) at 70% RH and under 21-h light conditions. Plants were then placed randomly on tables and positions rotated bi-weekly, and watered manually until October 17th, after which they were drip irrigated.

On November 14th, eight plants per genotype were randomly selected and exposed to the beetles inside a mousseline tent (area 4 m × 3; height 2 m). The rest of the plants (the controls) stayed outside the tent in the same room under similar growing conditions.

During the first week of December 2011 two leaves per plant were harvested and kept at 4 °C (maximum 4 days) before surface sterilisation. For practical reasons fungal morphotypes from the control plants were isolated first and thereafter from beetle-damaged plants. On the damaged plants, less damaged mature leaves were sampled from between six to eight replicates of each of the eight SwAsp genotypes under test ensuring that the leaves were of the same age and developmentally alike. Six leaf pieces per plant were then arranged on potato dextrose agar after sterilisation (PDA Merck KGaA, Germany).

*Experiment 2.* Due to the temporal sampling bias in the first experiment, a second experiment was initiated (July 10th 2012) to confirm that beetles might indeed cause an increase in endophyte richness and diversity. We used available small plants (~ 60 cm tall) generated from SwAsp tissue culture (genotype 18 initially hosting, and genotype 65 initially not hosting endophytes). These plants were individually covered with fine meshed mousseline fabric (height ~ 80 cm and diameter ~ 15 cm) and three randomly chosen plants per genet were subjected to beetle feeding (two beetles per plant). The first isolation event of fungal endophytes took place on August 1st 2012.

### Foliar chemical analyses

As part of *Experiment 1*, at the age of 5 weeks when the plants were ca. 70 cm tall the first fully expanded leaf from five randomly selected control plants per genet was harvested immediately into liquid nitrogen for salicinoid analyses. Leaves were lyophilised and stored at − 20 °C until they were ground to powder using a Retsch bead mill (Retsch GmBH, Haan, Germany). Leaf sample extracts were prepared and salicinoids quantified in the samples following methods described in Abreu et al. (2011). Briefly, 10.00 ± 1.00 mg ground leaf tissue was extracted in 1 ml methanol:chloroform:water (v:v:v) at 4 °C. Deuterated salicylic acid [^2^H_6_] (Isotec, Miamisburg, USA) was included as an internal standard in all samples. Samples were centrifuged at 4 °C and 100 µl of the resulting supernatant was vacuum dried. Prior to analysis, each dried sample was resuspended in 25 µl of methanol and 25 µl of 0.1% v/v aqueous formic acid. Injections of 2 µl of each sample were separated on a C18 UPLC™ column (2.1 × 100 mm, 1.7 µm) and analysed with a LCT Premier TOF/MS in negative mode (Waters, Milford, MA, USA), as described by Abreu et al. (2011). The following standard compounds were included in the analyses: salicin (Sigma-Aldrich, Stockholm, Sweden); cinnamoyl-salicortin (Keefover-Ring et al. 2014) and tremulacin were isolated from aspen at Umeå Plant Science Centre, and salicortin, tremuloidin and HCH–salicortin were provided by Prof. Lindroth (University of Wisconsin, USA). Single-ion chromatograms were extracted using MassLynx 4.1 software package (Waters Corp., USA) and salicinoids were quantified from single-ion chromatographic peaks using an in-house script in the Matlab environment at the Swedish Metabolomics Centre (SMC, Umeå, Sweden). Peak areas normalised by extracted sample weight and salicinoid compounds were identified from analytical standards and masses of either or both of the deprotonated ion ([M−H]−) and the formate adduct ([M−H + FA]−), based on molecular weights according to Abreu et al. (2011) and Keefover-Ring et al. (2014) and guided by retention times where available.

### Competition observation and experimentation

During the isolation event, evidence of interaction between common morphotypes was evaluated through growth measurements. After isolation, all plates contained filamentous morphs whereas yeasts only appeared on some. Thus, the first growth assessment of filamentous fungi could be related to random growth of yeasts from the isolation event. To investigate fungal interactions further, a competition experiment was setup with two plant-related filamentous morphotypes together with two beetle-related yeasts. In a competition experiment, inoculums of one filamentous and one yeast fungal isolate were then placed 2.5 cm apart on plates, and single morphs inoculated together as controls. Initially, five plates were setup for each yeast–filamentous fungi combination, thereafter an additional 40 plates were arranged with combinations of fungi for which additional replication (with 40 plates) promised significant insight (according to power analyses and calculations of least significant sample numbers, LSN, Table 3). Colony sizes were assessed using ImageJ (an open source image processing programme https://imagej.net/Welcome).

### Fungal DNA extraction and sequencing

Fungal mycelium from colonies that had been selected to get the most complete coverage of the diverse fungal material was carefully scraped from the PDA plates and ground in 2-ml Ep-tubes in liquid nitrogen. DNA extraction was performed with “E-Z 96 fungal DNA kit” (Qiagen) following the manufacture’s protocol. Nano-drop spectrometry was used to establish DNA yield and quality, and all DNA samples with a 260/280 ratio higher than 1.8 were used for the following PCR steps (Thermo scientific). Oligos were synthesised in Cybergene (http://www.cybergene.se) including: ITS-F (CTTGGTCATTTAGAGGAAGTAA) paired with ITS-R (TCCTCCGCTTATTGATATGC), and nuSSU-F (TTAGCATGGAATAATRRAATAGGA) paired with nuSSU-R (TCTGGACCTGGTGAGTTTCC).

Each PCR reaction mixture contained 50 μl of 10xPCR buffer, 1 μM primer, 0.2 mM dNTPs, 1.5 mM MgCl2, 1.25 U Taq polymerase and 1 ng/μl fungal DNA. PCR steps were programmed as 95 °C for 5 min, 33 cycles of 95 °C for 30 s, 51 °C for 30 s, 72 °C for 30 s and 72 °C for 10 min. PCR products were kept at 4 °C before analysing via gel electrophoresis on 1.5% Agarose (BIO-RAD) gel and visualising under UV light with 0.1% GelRed (GelRedTM Nucleic Acid Gel Stain, 10,000X in DMSO). PCR products were purified when specific bands were detected, using QIAquick PCR purification kit (Qiagen). The purified PCR products were delivered to Eurofins/mwg operon (http://ecom2.mwgdna.com) for sequencing. The resulting sequence data were blasted against the NCBI database (http://www.ncbi.nlm.nih.gov/) and against the yeast genome database (http://www.yeastgenome.org/cgi-bin/blast-fungal.pl). The search standard was “nucleotide collection nr/nt”. No filtre was used and the best blast hit and maximum percentage of matching (> 95%) for a ITS region was used as a standard to identify fungal taxon as presented in (ESM2).

### Statistics

Likelihood ratio tests were used to compare fungal composition, and together with *t* tests and ANOVA models analysed with the software JMP or R (R Core Team 2013). Simpson’s Index was calculated to compare the diversity of endophyte morphotypes. The Simpson’s index is based on *D* = sum p_i^2 (where p_i is the proportional abundance of species *i*); thus, the index increases with decreasing diversity. The Simpson’s Index was calculated for each individual plant using the R package “vegan” (Oksanen et al. 2017), and averages were calculated per genotype. Bipartite interaction matrices were calculated, and bipartite network interactions visualised, using the bipartite package in R (R Core Team 2013).

## Results

The total concentration of salicinoids in the leaves was 184–378 mg/g DW depending on genet (Table 1). Fifteen salicinoids were isolated from the chromatograms based on peak area when isomers of cinnamoyl-containing compounds were integrated together as suggested by Abreu et al. (2011) and Keefover-Ring et al. (2014). After isolation, morphotypes were characterised based on colour, texture and growth (Table 2). No significant relationship was detected between the mean values of total salicinoids of the control plants and the number of fungal morphotypes (ESM3). Fungal richness could also not be related to any specific salicinoid profile (ESM1). The best fit between any average concentration of an individual salicinoid in the control leaves and the number of fungal species was detected for acetylsalicortin (ANOVA, *R*_2_ = 0.09; *F*_7.1_ = 0.62; *P* < 0.46). The significance was not improved by separating the effect of CN genets (*N* = 4) from TL genets (*N* = 4), although the explanatory value substantially increased for that model (ANOVA, *R*_2_ = 0.41; *F*_7.1_ = 0.91; *P* < 0.51).

**Table 1:** Aspen (*P. tremula*) genets were propagated from the Swedish Aspen collection (SwAsp, Luquez et al. 2008)

Genotype	Salicortin	Tremulacin	2′Cin.Sal.	AcetylSal.	HCH_Sal.	Total
7	122.6 ± 7.5	63.0 ± 4.1	0.1 ± 0.0	1.7 ± 0.1	27.5 ± 2.7	249.3 ± 10.5
14	176.0 ± 10.2	15.3 ± 2.0	9.1 ± 0.5	2.2 ± 0.2	81.6 ± 15.2	327.4 ± 20.8
18	157.5 ± 12.1	108.2 ± 13.7	0.1 ± 0.0	2.5 ± 0.2	75.1 ± 14.2	377.9 ± 14.6
23	162.5 ± 4.3	29.7 ± 1.9	8.4 ± 0.6	2.8 ± 0.1	25.3 ± 5.9	274.1 ± 7.7
52	105.3 ± 6.3	19.0 ± 1.4	6.6 ± 0.3	1.3 ± 0.1	13.3 ± 2.3	183.6 ± 8.4
60	160.2 ± 14.6	83.9 ± 5.0	0.1 ± 0.0	2.4 ± 0.1	26.8 ± 8.1	312.7 ± 24.1
65	117.2 ± 11.8	6.1 ± 0.9	6.8 ± 0.8	0.7 ± 0.1	41.5 ± 5.7	218.2 ± 17.5
100	172.5 ± 10.6	88.8 ± 4.1	0.1 ± 0.0	2.0 ± 0.1	25.6 ± 4.5	327.0 ± 5.6

Average salicinoids content in the aspen genets of the experiment (mean ± SE in mg per g leaf DW, *N* = 5) are presented by genotype. The entire list of 15 salicinoids are detailed in ESM1 for all plant replicates

*Cin.* Cinnamoyl, *Sal.* Salicortin, *HCH* hydroxy-6-oxo-2-cyclohexene

**Table 2:** An overview of the fungi that were isolated for this study after tissue origin and putative taxon for ITS sequenced samples (please see SEM1 for a detailed list)

Tissue	*N*	Kind	Description	Putative taxon	ID competition
Leaves, undamaged	8	Fi	blu–gre, ***S***	n.a.	
		Fi	gry–blu, ***F***, sp	*Cladosporium* sp.	
		Fi	gry–blk, ***F***, sp	Pezizomycotina	
		Fi	gry–gre, ***F***, sp	*Penicillium brevicompactum*	ID: A4 = colony E
		Fi	or–yl, ***F***	Hypocreales	
Leaves, damaged	20	Fi	blk, ***S***	*Cladosporium cladosporioides*	
		Fi	blu–gre, ***F***	n.a.	
		Fi	brw, ***S***, po	n.a.	
		Fi	gre–blk/or, ***F***	*Penicillium*	
		Fi	gry–blu, ***S***	*Penicillium* sp.	
		Fi	gry–gre/gre, ***F***	*Penicillium expansum*	ID: B4 = colony C
		Fi	wh/gre, ***F***	*Arthrinium*	
		Fi	wh–blk, ***S***	n..a.	
		Fi	wh–blk, ***F***	Trichocomaceae	
		Y	Wh, ***F***	*Cryptococcus* sp.	ID: C10 = colony S
		Fi	wh, ***S***	n.a.	
		Y	yl–wh, ***F***	Basidiomycota	ID: C1 = colony L
Beetle	17	Y	R	*Rhodotorula* sp.	
		Fi	***F***	*Trichoderma*	

*N* indicates the number of successfully sequenced morphotypes of fungi for a certain tissue type: aspen (*P. tremula*) leaves grown in the absence (controls) or in presence (damaged) of *C. tremula* leaf beetles for 15 days. Beetle-associated fungi are included under “Beetle” Tissue. Kind distinguishes the isolates after appearance as either filamentous- or yeast-like, and a short hand description is included with codes appearing at the bottom of the table. ID Competition details the origin and putative taxon of the isolates that were used to test interactive strength in the competition experiments

Kind of morph: *Fi* filamentous, *Y* yeast-like; Description, colour: *blk* black, *blu* blue, *brw* brown, *gre* green, *gry* grey, *or* orange, *r* red, *wh* white, *yl* yellow; Growth development: *F* fast, *S* slow, additional surface characteristics: *sp* sporulating, *po* powdery, *t* transparent, *f* fluffy, *m* milky. ID for isolates that were used in the competition experiment, corresponds to ESM2

### Determination of fungi

In total, 86 plates representing 22 morphotypes from over 250 leaf segments were collected from experiments 1 and 2, in addition to eight morphotypes that were directly isolated from the beetles. Eight genera and 14 distinct species were determined on the basis of nucleotide matching in the NCBI database (http://www.ncbi.nlm.nih.gov/) and in the yeast genome database (http://www.yeastgenome.org/cgi-bin/blast-fungal.pl (Table 2).

### Fungal relationship with host genotype

Four fungal morphotypes were isolated from control plants in experiment 1 (Table 2). Endophyte richness initially displayed a genotype-specific pattern (Likelihood ratio: *N* = 32, *df* = 7, *χ*^2^ = 18.643, *P* = 0.0094) with no fungi emerging from genotypes 65 and 23. The most abundant morphotype E (identified as *Penicillium brevicompactum*) grew on 25% of the plates.

From beetle-damaged plants, colonies emerged within 2 days on all plates and fourteen distinct fungal morphotypes were rescued and determined (Table 2). Abundance varied between 1–4 colonies per leaf piece, and unlike the control plants, the impact of genet could no longer be distinguished by the morphotypes that grew from beetle-chewed plant samples (ANOVA *F* (61, 7) = 0.83, *P* = 0.57). Specific fungal morphotypes were, however, associated with certain genotypes when analysed one by one: morphotype L (*Basidiomycota*) grew only from genotypes 14, 18, and 100 (Likelihood ratio: *N* = 62, *df* = 7, *χ*^2^ = 25.48, *P* = 0.0006). Morphotype ν (*Arthrinium phaeospermum*) was only isolated from the genotypes 18, 52, and 100 (Likelihood ratio: *N* = 62, *df* = 7, *χ*^2^ = 14.1, *P* = 0.049). Morphotype S (*Cryptococcus* sp.) was restricted to genotypes 100 and 65 but without any strong evidence of genotype preference (Likelihood ratio: *N* = 62, *df* = 7, *χ*^2^ = 12.29, *P* = 0.09). The most abundant morph, morphotype C (*P. expansum*) that emerged on 86% of the plates, did not show any genotype preference (Likelihood ratio: *N* = 62, *df* = 7, *χ*^2^ = 7, 96, *P* = 0.34).

### Host–endophyte relationships

**Fig. 1:**
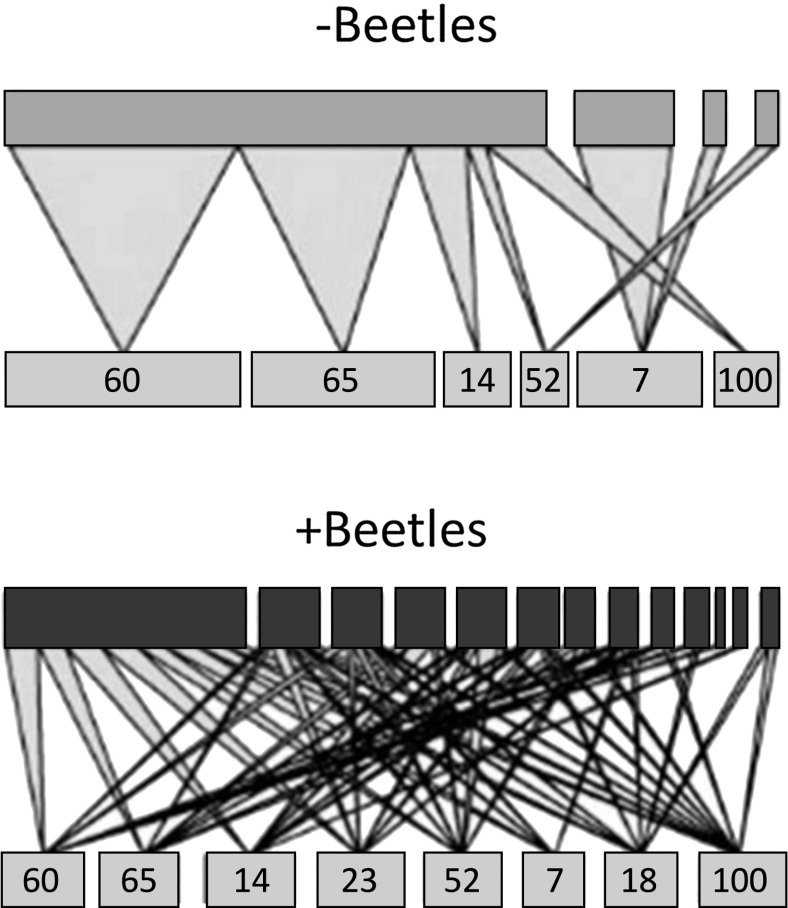
Bipartite graph of the relationship between the SwAsp genets and the endophyte community that they associated with, respectively, in absence (upper panel) and presence (lower panel) of *Chrysomela tremula* leaf beetles. Thicknesses of lines that connect genets with morphotypes are scaled to the abundance with a morphotype occurred in the samples. The fungal morphotypes are listed in Table 2

**Experiment 1.** Bipartite graphs were prepared to show how the mycobiome is related to genotype (Fig. 1). The number of links between hosts and endophytes increased in the presence of *Chrysomela* beetles, supporting the change from a simple relationship between genet and the associated endophyte community in the absence of the beetles, to a generalistic and more antagonistic web after beetles had interacted with the plants. Simpson indices, calculated per genotype, averaged 0.618 ± 0.142 (mean ± standard error) in controls and 0.339 ± 0.088 in beetle-damaged plants (*N* = 80). ANOVA analyses confirmed that endophyte diversity was significantly affected by aspen genotype (*F*_78,7_ = 3.9, *P* < 0.001**), beetle treatment (*F*_78,1_ = 19.7, *P* < 0.0001***), and their interaction (*F*_78,7_ = 3.1, *P* < 0.001**). These results also agree with the lower connectance values (calculated as the proportion of all possible links between genotype and endophyte richness) that were calculated for host and endophyte community and similar linkage density levels for control plants, as visualised in the bipartite webs (Fig. 1).

**Experiment 2.** We repeated the isolation experiment to secure simultaneous sampling of leaves from control and damaged plants. We used SwAsp clones 18 and 65 that at the time were available in the propagation facility at UPSC, Umeå. We expected more fungal morphotypes on beetle plants than on controls and, therefore, used a one-tailed *t* test to analyse the data. On average, we found 9 ± 1.08 fungal morphotypes on control trees (~ 2.54 colonies per segment) and 12.67 ± 1.38 morphotypes on damaged plants (1.8 colonies per segment). The difference in number of morphotypes per genotype was on average − 1.74 morphotypes per tree (Welsh *t* test, assuming unequal variances, one-tailed *t* = 2.09; *df* = 7.99; *P* = 0.035).

### Beetle-associated fungi and their interaction with the plant mycobiome

**Fig. 2:**
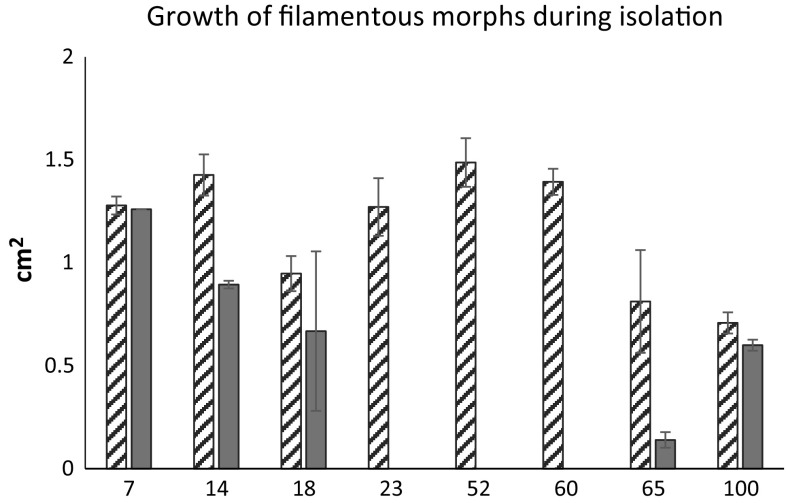
*Penicilium* sp. grew less vigorously when cultured from aspen leaves together with yeast colonies (average colony diameter, average size ± SE in cm^2^; for genet number 7, yeast was only appeared on one petri dish and no SE is included). Host trees had been propagated from the Swedish Aspen bio resource at Umeå Plant Science Centre and the numbers on *x*-axis refer to SwAsp genet number. Competition studies reported in Table 3 were setup to test the generality of the initial inhibition yeast appeared to have on growth of filamentous fungi during isolation

Yeast-like morphotypes were not observed in leaf tissues of control plants, but they were isolated from *Chrysomela*-damaged plants and were also isolated directly from the beetles. During the isolation, growth of filamentous fungi dominated the mycobiome of the beetle-damaged plants. Several of these isolates were later characterised as belonging to the *Penicillium* spp. During the isolation event, this morphotype on average grew to only 53.7% of its potential size when it co-occurred with yeast-like morphotypes such as *Basidiomycota* and *Cryptococcus* sp. (*t* test; *N* = 54; *df* = 52, *t* = − 5.36; *P* < 0.0001; *R*^2^ = 0.36, Fig. 2).

Morphotype E (*P. brevicompactum*) from the control plants and morphotype C (*P. expansum*) from the damaged plants were challenged in competition experiments with yeast-like morphotypes, L (*Basidiomycota*) and S (*Cryptococcus* sp.). The growth of the filamentous morph E line (*P. brevicompactum*) from the control plants appeared to be stimulated by both yeasts, but only significantly by yeast S. Filamentous morph C from damaged plants grew less in combination with yeast S (*Cryptococcus* sp.) than with yeast L (*Basidiomycota*). One yeast morph (L) was not affected by the presence of any of the filamentous morphs, whereas the other yeast (S) was generally limited by the filamentous fungi, mostly by C from the beetle-damaged plants. Power analysis suggested that it would demand more than 300 plates (least significant numbers) to verify the potential stimulating effect S had on E, and this combination was not repeated. An organism may theoretically affect another in three fundamentally different ways (positive, neutral, and negative) and the interaction outcome may then be described in terms of amensalism (−/0, as for C/L), commensalism (+/0, as for E/L), competition (−/−, as for C/S) and parasitism (± as for E/S) (as detailed in Table 3). No examples of neutralism (0/0) or mutualism (+/+) between the challenged fungi were detected in the competition experiments of this study.

**Table 3:** In a series of two experimental setups, interactions between the filamentous morphs (C ~ *Penicillium brevicompactum* and E ~ *Penicillium expansum*) were tested against the yeast-like morphs (L ~ *Basidiomycota* and S ~ *Cryptococcus*)

	Effect on C	*N*	Effect on E	*N*	Effect on L	*N*	Effect on S	*N*
Filamentous C					77.7%^*n.s.*^	40	2.6%**	40
Filamentous E					91.5%^*n.s.*^	5	50.1%*	5
Yeast L	85.7%**	40	249.3%**	5				
Yeast S	52.7%**	40	153.3%^*n.s.*^	5				

Pilot tests were setup with five replicates (*N*) and repeated when an effect was to be expected from enhancing the sample size to 40. The effect of the various combinations are listed for each morph by column, and values less than 100% means that growth was restricted by the co-existence, whereas a value higher than 100% indicates a stimulated growth effect. Significant values are indicated by asterisks; * at the 5% level, and ** at the 1% level, “~” refers to tentative determination of isolate after blasting of ITS sequences in the European Nucleotide Archive (http://www.ebi.ac.uk/ena) as also listed in ESM2

## Discussion

Here, we demonstrate a genotype-specific association between young aspen plants from the Swedish Aspen collection and the endophyte mycobiome they host. Salicinoid phenolic compounds attract *Chrysomela* leaf beetles but we found no relationship between endophytes and the salicinoid profiles of control plants. Herbivory by the leaf beetles led to richer fungal assemblages and increased abundance of leaf-associated endophytic fungi. This resulted in a less specialised endophyte community that could no longer be associated with host genotype. Yeast morphs could be related to the presence of beetles, but competition experiments did not point to any systematic interaction related to fungal origin, which could support the existence of a stable three-way association between plant genotype, herbivory and mycobiome composition.

Salicinoid phenolic glycosides are diverse in the Swedish Aspen collection because of functional groups attached to the glucose molecule in various configurations (Abreu et al. 2011; Keefover-Ring et al. 2014). Among salicinoids reported for *P. tremuloides*, more complex (higher molecular weight) compounds may determine toxicity and palatability mainly to generalist herbivores (Ayres et al. 1997; Donaldson and Lindroth 2007). Clausen et al. (1989), for example, found a greater reduction in herbivore survival and performance caused by tremulacin (compared to the less complex salicin) and suggested that the benzoyl group on the molecule may enhance the toxic effect of the HCH **(**hydroxy-6-oxo-2-cyclohexene) group. For fungal endophytes in cottonwood hybrids, Bailey et al. (2005) reported a negative effect of condensed tannins on endophyte richness and abundance, but they found no effect of foliar salicinoid compounds (salicortin and HCH–salicortin). Although this study comprised too few genotypes to disentangle a potential relationship with salicinoid diversity, we did not detect any correlation that suggested that any one of the sixteen salicinoids individually shaped the mycobiome, which was also not correlated with the sum of salicinoids (ESM2).

### Beetle-associated fungi and network consequences

*Chrysomela* leaf beetles are specialist herbivores, many of which sequester the salicinoids of salicaceous trees for their own defence (Pasteels et al. 1983; Termonia et al. 2001; Boland 2015). Endophytes may enter the tissues of their hosts through stomatal openings or through wounds and damaged cells (Wilson 2000). Insect vectors may help fungi to disperse between plants and facilitate colonisation of host tissues; disruption of the plant epidermis breaks the physical barrier to render the interior tissues accessible for spores and hyphae of the fungi. Chewing damage will further elicit a JA (jasmonic acid) response in the plant that can result in a lowered SA (salicylic acid) response, which is likely to weaken the pathogen defence pathway (Verhagen et al. 2004; Herre et al. 2007). The symptomless endophytes may interact in complex and strain-specific ways as shown by Navarro-Meléndez and Heil (2014) for lima beans with the endogenous levels of SA and JA and with the defence traits up- and downstream of these controlling defence hormones. In this study, we found that *Chrysomela* beetles enriched the leaf mycobiome with endophytic morphotypes that were not cultured from the control leaves. The abundance of fungal morphotypes also increased after herbivory. To ensure that the increase in richness and abundance was not an artefact caused by biased sampling, we repeated the experiment in a smaller setup where we harvested leaves from control and damaged plants simultaneously. We could then confirm that morphotype richness indeed increased after the plants had been exposed to herbivory.

### The isolated fungi

Three-way relationships that involve trees, insects and fungi are well established for wood-dwelling species (Graham 1967) in which the interactions between the insect and fungus may indeed range from mutualistic to antagonistic (Kopper et al. 2004). Interactions between insects and foliar endophytic fungi are less studied (Albrectsen and Witzell 2012), but there are indications that insects may actively avoid leaves with high abundance of endophytic fungi (Van Bael et al. 2009; Coblentz and Van Bael 2013), although this is not always the case (Faeth and Hammon 1996).

Phylloplane fungi may serve as biocontrol agents against fungal pathogens through competition, antibiosis, or parasitism, and among endophytes the more host-specific and vertically transferred they appear to be, the more likely they are to exist in a mutualistic relationship with the host (Albrectsen and Witzell 2012; Christian et al. 2017). The isolated fungal endophyte associations in this study included several filamentous fungi belonging to *Penicillium* and *Cladosporium* (characterised as dominant cosmopolitans, Christian et al. 2017). These taxa will often participate in processes affecting decay resistance or decomposition (Zambell and White 2017). Isolated yeasts included *Cryptococcus* sp. (from leaves) and *Rhodotorula* sp. (from beetles). *Cryptococcus* can be strong niche occupants and competitors that may out-compete selected pathogens including *Botrytis cinerea* that causes grey mould in fruits such as apples and strawberries (Zambell and White 2017). During the isolation event, we found signs of interference among the isolates of certain fungal morphotypes. We observed slower growth of filamentous morphs in the presence of non-filamentous morphs (Table 2). Many endosymbiotic fungi of insects are yeast-like (Vega and Dowd 2005) and they are known to be unevenly distributed in leaf tissues (Solis et al. 2015).

In particular, we looked for evidence of competitive relationships among isolates with diverse isolation histories. Under the assumption that mutualism favours higher host specificity compared to antagonism (Kawakita et al. 2010), network analyses may suggest which qualities exist in a bipartite community (Bascompte et al. 2006). We found that the mycobiome that had formed in the leaves after herbivory was less associated with the genetic background of the host, which thus suggests that the relationship between these young aspen trees and the endophytic mycobiome developed into an increasingly common and potentially antagonistic structure (Bascompte et al. 2006).

Competition studies confirmed unbalanced effects between the tested endophytes. The two yeasts that we used for competition tests both reduced *P. expansum* but stimulated growth of *P. brevicompactum.* One yeast (L) was never affected by competition, whereas the other (S) was strongly reduced in growth, suggesting a true competitive situation in which both parts were negatively affected (−/−). *P. brevicompactum* always appeared to benefit from yeast presence either without reducing its counterpart (L, as commensalist) or at the expense of its counterpart (S, as parasite). This small experiment of four species, that were challenged pairwise, thus suggested that interactions (also in the leaf endosphere) may be highly complex and that its outcome may highly depend on the involved fungal strains.

We observed examples of amensalism, commensalism, competition and parasitism not typical of potentially co-evolved relationships (Kawakita et al. 2010). Some studies also suggest that a community will adapt according to the order in which its members arrive, and succession order is a potential mechanism by which endophyte inoculation may interfere with pathogens and affect later development of diseases (Raghavendra and Newcombe 2013; Busby et al. 2016), with the operating mechanism being pre-empt competition.

### Choice of detection method

As the study of endophytes is method-dependent (Hyde and Soytong 2008; Unterseher and Schnittler 2009; Sun and Guo 2012), the exact composition of the mycobiota is almost impossible to identify. Culturing methods are labour-intensive and biased (Solis et al. 2015): the nutrient-rich PDA plates used in this study, for example, have a tendency to oversample fast-growing filamentous morphs. An exhausting screening with the use of metagenomics methods will also be biased, for example, by including epiphytes (e.g. Hyde and Soytong 2008). A dilution-to-extinction culturing method that eliminates the effect of fast-growing fungi can be adopted (Collado et al. 2007; Unterseher and Schnittler 2009; Siddique et al. 2017). This method increases the chance of detecting rare morphs, speed up handling time (Shokralla et al. 2012; Lindahl et al. 2013), and NGS or HTS (high-throughput sequencing) technology that generates millions of fungal reads may also simultaneously and precisely test multiple effectors (Siddique and Unterseher 2016; Siddique et al. 2017). Phenotype microarrays (Blumenstein et al. 2015) further provide complementary information about fungal-defined substrate requirements. These new techniques are still rather expensive to use and they are equipment demanding (Porras-Alfaro and Bayman 2011). Although new techniques refine our ability to define the mycobiome, culturing continues to be a cheap way to provide fundamental insights.

## Conclusion

Our study supports that the foliar endophytic mycobiome of aspen is potentially a highly diverse and dynamic interface, which may indeed be shaped by direct and indirect interactions, between herbivores and fungal associates, and between aspen genotypes and associated organisms. We found no evidence that foliar salicinoids shape the genotypic associations for this set of undamaged greenhouse-grown plants. However, we cannot exclude that associations are so complex that a larger number of host genotypes are needed to explore potential effects of salicinoid profiles. In future studies, substrate requirements and niche overlap by the use of microtiter plates (Blumenstein et al. 2015) could also resolve effects of specific phenolic compounds; however at present with the selection of fungi found in this study, we cannot directly relate any leaf phenolic property to the presence or performance of any particular member of the mycobiome.

## Electronic supplementary material

Below is the link to the electronic supplementary material.

Supplementary material 1 (DOCX 142 kb)

Supplementary material 2 Overview of isolates and ITS determination of the taxon of this study. Sequence information is stored in https://www.ebi.ac.uk/ena (XLSX 22 kb)

Supplementary material 3 (DOCX 14 kb)
