# Supplementary material for: Both plant genotype and herbivory shape aspen endophyte communities
Source: Oecologia. 2018 Mar 1;187(2):535–45. doi: 10.1007/s00442-018-4097-3 (PMC5997111; doi:10.1007/s00442-018-4097-3)
Supplement: Supplementary file 1 — Supplementary material 1 (DOCX 142 kb) [file 442_2018_4097_MOESM1_ESM.docx]

**Salicinoid compounds in *Populus tremula* leaf samples identified using ultra high-performance liquid chromatography (UHPLC) with UV and electro-spray ionization time-of-flight mass spectrometry (ESI-TOF/MS). Identification based on (a) comparison with analytical standard; b) measured exact mass [M-H + HCOOH]− mass to charge ratio (m/z), based on single measurements in negative mode; c) measured exact mass [M-H]- mass to charge ratio (m/z), based on single measurements in negative mode; § isomers 1 and 2 (see Keefover-Ring et al., 2014). (1) Salicinoids reported in Abreu et al. (2011); 2) Salicinoids reported in Keefover-Ring et al. (2014).**

**Values (in mg g^-1^ dry weight) of foliar salicinoids quantified in all control plants of *P. tremula* identified using ultra high-performance liquid chromatography (UHPLC) with UV and electro-spray ionization time-of-flight mass spectrometry (ESI-TOF/MS). Salicinoids quantitied against analytical standards: 1 Salicortin, 2 Tremulacin, 3 Salicin, 4 Tremuloidin, 5 HCH-salicortin, 6 2'Cinnamoylsalicortin.**
