# Supplementary material for: Both plant genotype and herbivory shape aspen endophyte communities
Source: Oecologia. 2018 Mar 1;187(2):535–45. doi: 10.1007/s00442-018-4097-3 (PMC5997111; doi:10.1007/s00442-018-4097-3)
Supplement: Supplementary file 3 — Supplementary material 3 (DOCX 14 kb) [file 442_2018_4097_MOESM3_ESM.docx]

ESM3. A lack of relationship between relative genet-specific salicinoid content in *P.tremula* genets (average of five control plants in mg g^-1^ DW Table 1) and the number of morphotypes (fungal richness) isolated from a genet in beetle damaged plants.
